# Gut Microbiota Dysbiosis Is Associated with Inflammation and Bacterial Translocation in Mice with CCl_4_-Induced Fibrosis

**DOI:** 10.1371/journal.pone.0023037

**Published:** 2011-07-29

**Authors:** Isabel Gómez-Hurtado, Arlette Santacruz, Gloria Peiró, Pedro Zapater, Ana Gutiérrez, Miguel Pérez-Mateo, Yolanda Sanz, Rubén Francés

**Affiliations:** 1 Unidad Hepática, Hospital General Universitario, Alicante, Spain; 2 CIBERehd, Instituto de Salud Carlos III, Madrid, Spain; 3 Instituto de Agroquímica y Tecnología de Alimentos (IATA), Consejo Superior de Investigaciones Científicas (CSIC), Valencia, Spain; 4 Unidad de Investigación, Hospital General Universitario, Alicante, Spain; La Jolla Institute of Allergy and Immunology, United States of America

## Abstract

**Background:**

Gut is the major source of endogenous bacteria causing infections in advanced cirrhosis. Intestinal barrier dysfunction has been described in cirrhosis and account for an increased bacterial translocation rate.

**Hypothesis and Aims:**

We hypothesize that microbiota composition may be affected and change along with the induction of experimental cirrhosis, affecting the inflammatory response.

**Animals and Methods:**

Progressive liver damage was induced in Balb/c mice by weight-controlled oral administration of carbon tetrachloride. Laparotomies were performed at weeks 6, 10, 13 and 16 in a subgroup of treated mice (n = 6/week) and control animals (n = 4/week). Liver tissue specimens, mesenteric lymph nodes, intestinal content and blood were collected at laparotomies. Fibrosis grade, pro-fibrogenic genes expression, gut bacterial composition, bacterial translocation, host's specific butyrate-receptor GPR-43 and serum cytokine levels were measured.

**Results:**

Expression of pro-fibrogenic markers was significantly increased compared with control animals and correlated with the accumulated dose of carbon tetrachloride. Bacterial translocation episodes were less frequent in control mice than in treated animals. Gram-positive anaerobic *Clostridia* spp count was decreased in treated mice compared with control animals and with other gut common bacterial species, altering the aerobic/anaerobic ratio. This fact was associated with a decreased gene expression of GPR43 in neutrophils of treated mice and inversely correlated with TNF-alpha and IL-6 up-regulation in serum of treated mice along the study protocol. This pro-inflammatory scenario favoured blood bacterial translocation in treated animals, showing the highest bacterial translocation rate and aerobic/anaerobic ratio at the same weeks.

**Conclusions:**

Gut microbiota alterations are associated with the development of an inflammatory environment, fibrosis progression and bacterial translocation in carbon tetrachloride-treated mice.

## Introduction

Gut microbiota is a large and diverse population of living microorganisms highly susceptible to environmental and pathophysiological alterations [1]. It exerts nutritional, protective and trophic effects on the intestinal epithelium and immune system, maintaining symbiotic mutualistic interactions with the host [2], [3]. The gut microbiota is also a dynamic ecosystem that modifies its composition and physiological functions in response to severe habitat insults. Original studies on mice showed that gastrointestinal microbiota is mainly composed by *Enterobacteriaceae*, *Lactobacillus, Bacteroides* and *Clostridium* [4]; [5]. Some *Clostridium* clusters are involved in the production of short chain fatty acids like butyrate used as fuel for host's colonocytes [6]. In addition, butyrate has anti-inflammatory effects that result from inhibition of transcription factor NF-κB leading to a decreased secretion of pro-inflammatory cytokines [7]–[9]. Other bacterial groups such as *Bifidobacterium* also seem to be implicated in host's protection by preventing increased permeability and promoting a healthier microvillus environment [10], [11].

Experimental chronic liver damage is commonly associated with a several-fold increase of bacterial translocation (BT) [12], [13]. BT is a common and recurrent event occurring in cirrhosis and constitutes the current pathogenic theory for the onset of bacterial infections in this setting [14]–[16]. Intestinal bacterial overgrowth, increased permeability of the intestinal mucosal barrier, and deficiencies in local host immune defences are the major mechanisms postulated to favour BT in cirrhosis [17], [18]. Any of these abnormalities deranges clearance of endogenous bacteria from portal and systemic circulation, turning the gut into the major source of bacteria in life-threatening bacterial infections such as spontaneous bacterial peritonitis (SBP) [19].

We therefore hypothesize that microbiota composition may be affected and change along with the progressive liver damage induced in mice by oral administration of carbon tetrachloride (CCl_4_). From this point, the aim of this study has been to provide integrative data on inflammatory consequences associated with alterations in the gut microbiota composition and bacterial translocation, defined by presence of bacterial DNA in MLNs and blood [13], in mice with advanced CCl_4_-induced liver damage.

## Results

### Characteristics of animals and progressive liver injury

**Figure 1:**
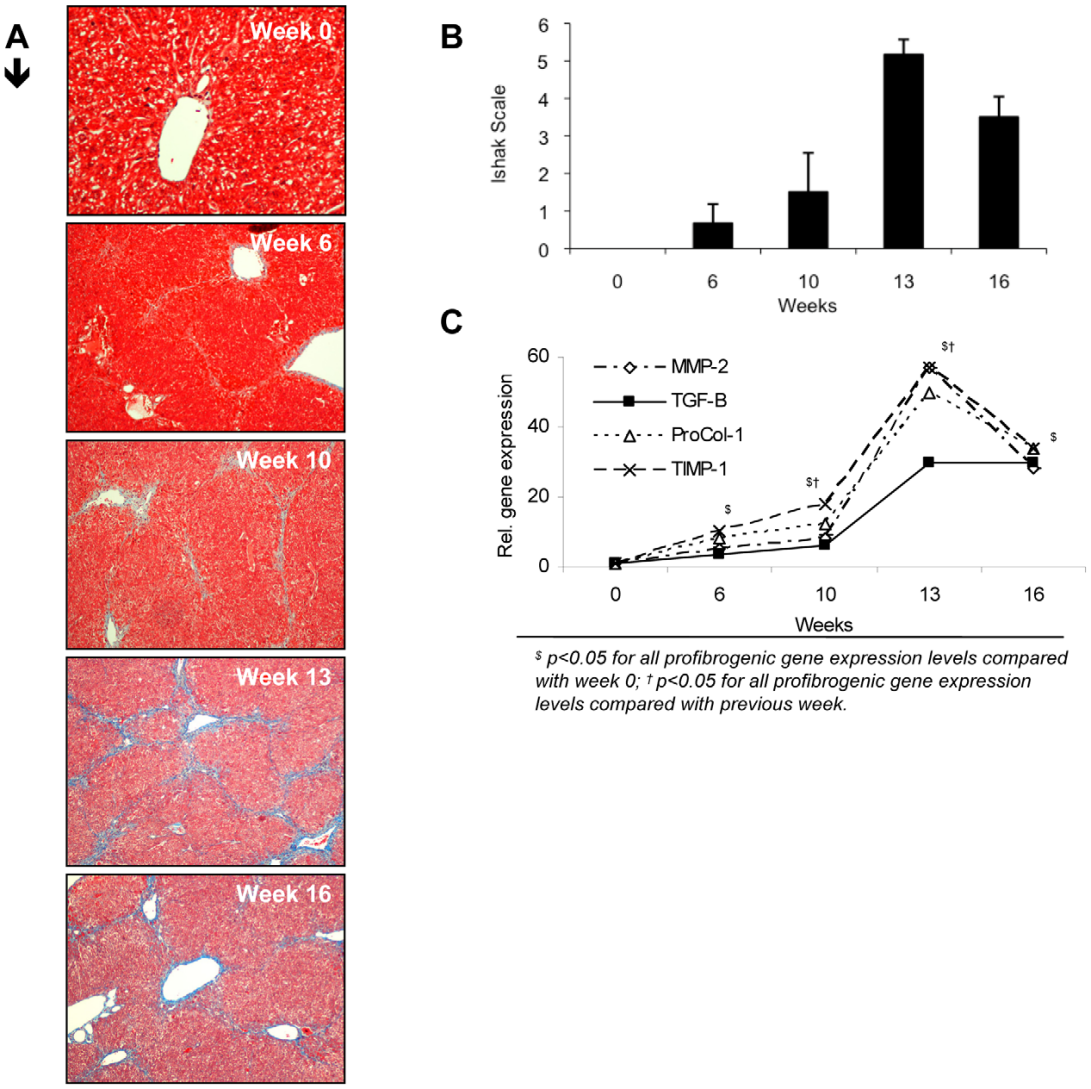
Fibrosis grade at laparotomy weeks along the study protocol in CCl4-treated mice. (A) Example of histological changes in CCl4-treated mice along the 16 weeks of study. Hepatic sections stained with Masson Trichrome (original magnification ×10), showing light to strong blue staining, as a reflection of the collagen deposition at different stages of the liver disease: no histopathological changes (week 0); focal mild portal fibrosis with short fibrous septa (week 6); moderate collagen fibber deposition in portal areas with occasional portal bridging (week 10); marked fibrosis in the majority of portal spaces with frequent portal-portal and portal-central bridging and architectural distortion (regenerative nodules) (weeks 13 and 16). (B) Fibrosis grade according to Ishak Scale in treated animals (n = 6/week) at different study weeks are represented as mean ± standard deviation. (C) Relative gene expression levels of different profibrogenic genes at different study weeks. MMP-2: Matrix metalloproteinase 2; TGF-β. Tumour growth factor beta; ProCol-1: Procollagen alpha-1(1); TIMP-1: Tissue inhibitor of metalloproteinase 1. Mean values of CCl4-treated mice (n = 6/week) are represented.

A total of 75 animals were included in the study. Fifty of them were weekly treated with CCl_4_ (see methods) and 25 mice constituted the control group. Twenty animals (40%) died during liver damage induction (treated group). None of the control animals died during the study protocol. Characteristics of control and treated mice at weeks of laparotomy, histopathological progressive liver damage induced and the evolution of profibrogenic markers during the study protocol can be followed in Table 1 and Figure 1, respectively. Regenerative nodules in treated animals were considered as evidences of cirrhosis. Expression of all profibrogenic markers was significantly increased from week 6 when compared with levels in non-treated mice. The highest relative expression of all profibrogenic markers and the most severe liver fibrosis happened at week 13. Accumulated dose of CCl_4_ correlated with relative gene expression of all profibrogenic markers (*vs* TGF-B, r = 0.883; *vs* MMP-2, r = 0.887; *vs* ProCol-1, r = 0.893; *vs* TIMP-1, r = 0.883; *p*<0.001 in all cases).

**Table 1:** Characteristics of animals at laparotomies.

	week 0			week 6						week 10						week 13						week 16					
	control (n = 6)			control (n = 4)			CCl4 (n = 6)			control (n = 4)			CCl4 (n = 6)			control (n = 4)			CCl4 (n = 6)			control (n = 4)			CCl4 (n = 6)		
Body weight (gr)	19,80	±	0,30	21,90	±	1,26	21,12	±	0,79	22,62	±	1,05	21,84	±	1,76	22,17	±	0,74	22,88	±	1,24	23,28	±	0,55	21,16	±	1,97
Liver weight (gr)	0,92	±	0,12	1,01	±	0,12	1,46	±	0,38	1,00	±	0,09	1,11	±	0,11	0,89	±	0,06	1,22	±	0,08 *	1,04	±	0,05	1,52	±	0,15 *
Spleen weight (gr)	0,08	±	0,03	0,11	±	0,01	0,18	±	0,06 *	0,10	±	0,03	0,13	±	0,01	0,10	±	0,02	0,13	±	0,01 *	0,11	±	0,02	0,13	±	0,03
10cm ileum content (gr)	0,14	±	0,02	0,09	±	0,02	0,12	±	0,07	0,08	±	0,06	0,03	±	0,01 *	0,06	±	0,02	0,03	±	0,01 *	0,07	±	0,01	0,09	±	0,02
Total cecum content (gr)	0,19	±	0,04	0,16	±	0,03	0,20	±	0,06	0,11	±	0,03	0,10	±	0,45	0,16	±	0,04	0,13	±	0,06	0,18	±	0,06	0,32	±	0,09
Accum CCl_4_ dosage (uL)		−			−		435,5	±	34,90		−		798,0	±	87,65		−		1049	±	73,49		−		1647	±	130,8

*p<0,05 compared with the weekly control group.

### Alterations in gut microbiota content during induction of cirrhosis are associated with blood inflammatory events

**Figure 2:**
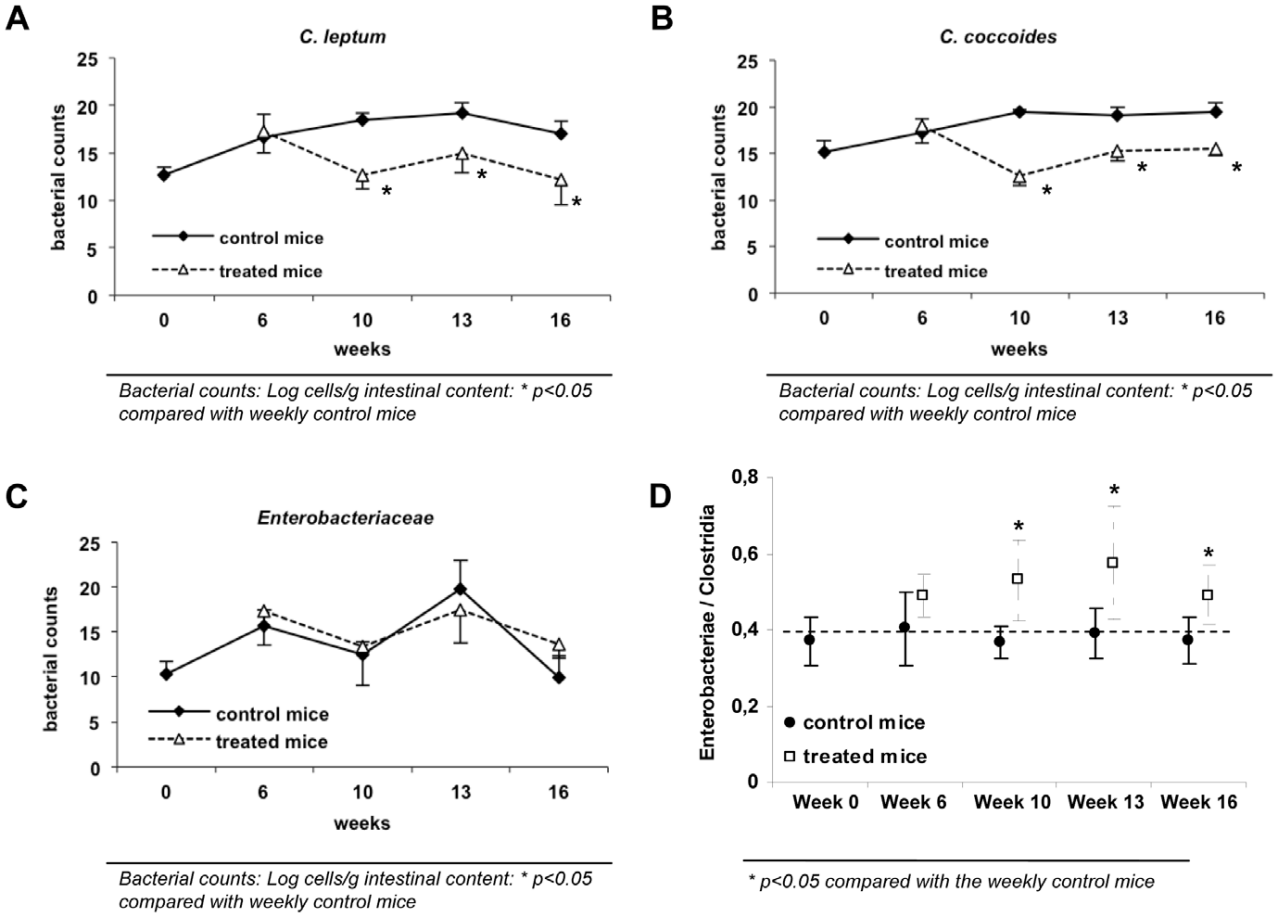
Temporal evolution of *Clostridium* groups and *Enterobacteriaceae* counts and their ratio in control and CCl_4_-treated mice. (A, B, C) Bacterial counts from samples of cecum content were measured by quantitative real-time PCR and represented as *Log* cell/g intestinal content. Values shown as mean ± standard deviation are represented for control (n = 4/week) and treated mice (n = 6/week) along the 16 weeks of study. (D) Differences in the *Enterobacteriaceae/Clostridia* ratio are represented as mean ± standard deviation for control (n = 4/week) and treated mice (n = 6/week) along the 16 weeks of study.

The effect of continuous administration of CCl_4_ to mice on gut microbiota composition was evaluated and compared with control animals at several stages during the study protocol (Table 2). As can be observed, no statistical differences were detected for *Enterobacteriaceae*, *Lactobacillus* group and *Bacteroides fragilis* group between control and treated animals at different weeks. In the case of the *Bifidobacterium* genus, despite initial differences in concentration between control and treated mice, its numbers became very similar from week 10 on. However, numbers of *Clostridium leptum* group and *Clostridium coccoides* group were significantly reduced in treated animals compared with control mice from week 6 to the end of the study time-frame. Figure 2 shows the ratio of *Enterobacteriaceae* to *Clostridium* groups in control and treated mice, as an index of the proportion of aerobic bacteria in relation to anaerobic bacteria in the intestinal contents. Whereas this ratio was constant along the study protocol for control animals, it significantly increased in treated mice along the study weeks.

**Table 2:** Gut microbiota changes during the study protocol in control and CCl_4_-treated mice.

	week 0			week 6						week 10						week 13						week 16					
Bacterial cluster (counts)	control (n = 6)			control (n = 4)			CCl4 (n = 6)			control (n = 4)			CCl4 (n = 6)			control (n = 4)			CCl4 (n = 6)			control (n = 4)			CCl4 (n = 6)		
*Enterobacteriae*	10,3	±	1,34	15,66	±	1,74	17,29	±	3,74	12,45	±	1,42	13,38	±	4,32	19,78	±	3,19	17,43	±	3,63	9,89	±	2,49	13,62	±	1,52
*Lactobacillus*	15,7	±	0,83	16,71	±	0,44	17,56	±	1,34	16,55	±	1,24	14,49	±	1,89	16,22	±	1,47	17,88	±	1,04	16,24	±	0,95	14,23	±	1,12
*Bacteroides*	12,7	±	0,96	15,76	±	2,49	18,33	±	1,84	17,10	±	0,60	16,47	±	1,09	19,10	±	1,52	18,50	±	1,39	14,57	±	1,28	15,94	±	0,98
*Bifidus*	10,1	±	2,13	7,91	±	0,88	12,82	±	1,04 *	17,00	±	2,42	13,13	±	1,73 *	13,84	±	0,41	13,45	±	1,36	12,66	±	1,10	14,70	±	2,33
*C. leptum*	12,7	±	0,82	16,64	±	2,43	17,25	±	2,24	18,48	±	0,71	12,65	±	1,43 *	19,24	±	1,04	14,99	±	2,06 *	16,98	±	1,41	12,20	±	2,64 *
*C. coccoide*	15,2	±	1,16	17,19	±	1,51	17,94	±	1,87	19,52	±	0,25	12,61	±	1,06 *	19,12	±	0,78	15,26	±	1,05 *	19,52	±	0,99	15,52	±	0,79 *

*Bacterial counts: Log cells/g intestinal content:*

** p<0.05 compared with weekly control mice.

**Figure 3:**
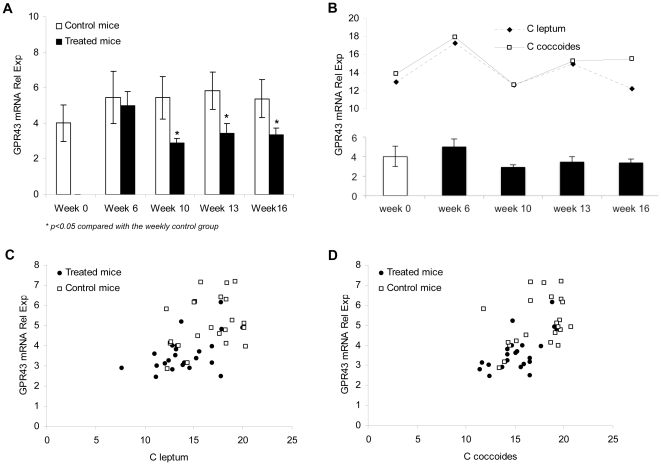
Specific G protein-coupled receptor (GPR)-43 gene expression along the study protocol. (A) Temporal mRNA expression of GPR-43 in PMN cells of treated and control mice. Values were measured by quantitative real-time PCR and are shown as mean ± standard deviation for control (n = 4/week) and treated mice (n = 6/week) along the 16 weeks of study. (B) Relative mRNA expression of GPR-43 PMN levels, and *C. leptum* and *C. coccoides* counts from samples of intestinal cecum content in treated animals (n = 6/week) are shown at different laparotomy weeks. (C, D) Correlation between GPR-43 PMN gene expression levels and *C. leptum* (C) or *C. coccoide* (D) counts in all control (n = 22) and treated mice (n = 24) included in the study.

To determine a possible relationship between inflammation and decreased *Clostridium* numbers in the intestine of treated mice, temporal mRNA expression of butyrate specific G protein-coupled receptor (GPR)-43 was analyzed in PMN cells of treated and control mice. Figure 3 shows a statistically significant GPR43 decrease in cells from CCl_4_-treated mice compared with control mice from week 10, at which the lowest levels of GPR43 are observed. As can be followed, variations in GPR43 relative mRNA expression are similar as those of *Clostridium* group numbers along the weeks. In fact, a positive correlation was obtained between GPR43 relative gene expression levels and the *Clostridium* group numbers when the total series of mice included in the study were considered.

**Figure 4:**
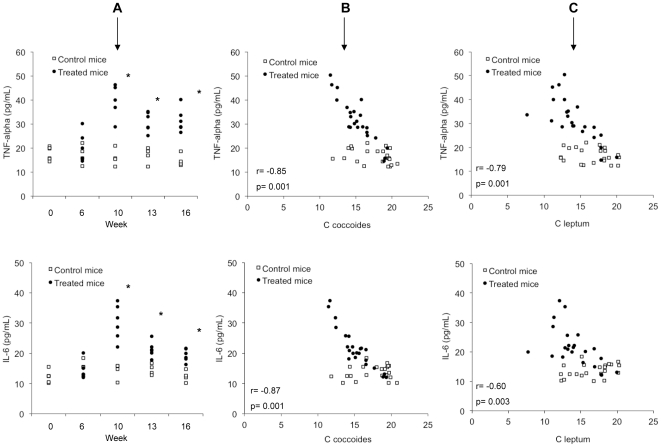
Pro-inflammatory cytokine profile along the study protocol. (A) TNF-α and IL-6 serum levels in control (n = 4/week) and treated mice (n = 6/week). Values, measured by cytometric bead arrays, obtained from each single mouse at different study weeks are represented in the Figure. *p<0.05 compared with the weekly control group. (B) Correlation between serum TNF-α and IL-6 with *C. coccoides* counts from samples of intestinal cecum content in all control (n = 22) and treated mice (n = 24) included in the study. (C) Correlation between serum TNF-α and IL-6 with *C. leptum* counts from samples of intestinal cecum content in all control (n = 22) and treated mice (n = 24) included in the study.

Pro-inflammatory cytokines TNF-α and IL-6 were also evaluated in blood of treated and control mice. Treated mice showed significantly higher levels of TNF-α and IL-6 at every week of study compared with control mice, reporting the highest levels of both cytokines at week 10 (Figure 4A). Serum levels of these cytokines inversely correlated with the concentration of *C. coccoides* group and *C. leptum* group, as can be observed in Figure 4, and with GPR43 relative gene expression (r = −0.72, p = 0.01 in the case of TNF-α; and r = -0.78, p = 0.01 in the case of IL-6).

### Rate of bacterial translocation episodes is associated with gut dysbiosis and liver damage progression

BT, defined by presence of bacterial DNA in MLNs and blood [13], was evaluated at laparotomies along the study protocol (Table 3). Overall, bacterial DNA translocation was more frequently present in treated animals (14/24, 58.3%) compared with control mice (3/22, 13.6%, *p*<0.05). All animals with bacterial DNA in MLNs except for one in the treated group showed simultaneous presence of the corresponding bacterial DNA in blood. Sequencing analysis identified *Escherichia coli* in 10 samples (8 from treated and 2 from control mice), *Streptococcus pneumoniae* in 3 samples (2 from treated and 1 from control mice), and *Staphylococcus aureus* in 2 samples, *Shigella flexneri* in 1 sample and *Campylobacter jejuni* in 1 sample from treated mice. This last species corresponded to the single discrepancy between bacterial DNA identified in MLNs and not in blood. The use of species-specific primers in MLN samples corroborated first, the presence of the sequenced bacterial DNA and, second, the absence of other simultaneous species.

**Table 3:** Bacterial DNA translocation at laparotomies in control and treated mice.

	Control mice (n = 22)		Treated mice (n = 24)	
Week	MLNs	Blood	MLNs	Blood
0	0/6 (0%)	0/6 (0%)	−	−
6	1/4 (25%)	1/4 (25%)	1/6 (17%)	1/6 (17%)
10	0/4 (0%)	0/4 (0%)	3/6 (50%) *	2/6 (33%) *
13	1/4 (25%)	1/4 (25%)	5/6 (83%) *	5/6 (83%) *
16	1/4 (25%)	1/4 (25%)	5/6 (83%) *	5/6 (83%) *

*p<0,05 compared with the weekly control group; MLNs: mesenteric lymph nodes.

BT rate in control mice was not increased along the study weeks. However, in the group of treated animals, percentage of BT episodes was incremented at weeks 13 and 16 along with fibrosis grade, from 17% of BT present in non-fibrotic mice up to more than 80% of mice with the most severe fibrotic grades, and with gut dysbiosis, reaching the highest rate at the same week aerobic/anaerobic ratio was higher. Serum levels of both cytokines were significantly higher in animals with BT compared with non-BT mice, independently of the study week (TNF-α, 29.0±8.1 pg/mL *vs* 21.5±8.1 pg/mL, *p*<0.05; IL-6, 20.8±3.8 pg/mL *vs* 15.8±3.4 pg/mL, *p*<0.05).

## Discussion

The present study shows that a decrease in *Clostridium* group numbers in gut microbiota content is associated with an increased inflammatory response and liver injury progress in CCl_4_-treated mice. This dysbiosis is, in turn, timely associated with and increased rate of bacterial DNA translocation episodes in treated mice, suggesting a key role for gut microbiota changes in the development of BT during induction of experimental cirrhosis.

Cirrhosis represents the end-stage of any chronic liver disease, characterized by the most advanced stage of fibrosis, distortion of the liver parenchyma associated with septae and nodule formation, altered blood flow and the potential development of liver failure at long term. Bacterial infections are common complications in patients with decompensated cirrhosis and are thought to happen by deficiencies in the host immune defences, impairment in permeability of the intestinal mucosal barrier and/or intestinal bacterial overgrowth [17], [18]. All these factors are postulated to favour BT in advanced cirrhosis, which has already been demonstrated in experimental cirrhosis [13], [20]. As all suggested BT mechanisms are related to alterations in the intestinal microbiota, which constitutes the major source of endogenous bacteria, in the present work we have evaluated the quantitative changes in the main clusters of gut microbiota in mice and the associated inflammatory status along the induction of experimental cirrhosis in the final outcome of an increased BT risk.

Mice treated with weight-controlled CCl_4_ increasing amounts were followed for 16 weeks and compared with control mice, as described in Methods section. During this period, expression of pro-fibrogenic markers was significantly increased compared with control animals, and correlated with the accumulated dose of CCl_4_. As expected, BT episodes were less frequent in control mice than in treated animals and, among them, BT incidence increased as fibrosis grade did. But one of the most relevant findings in this investigation was that, in this time-frame, the numbers of Gram-positive anaerobic *Clostridium* groups decreased in treated mice compared with control animals and with other gut common bacterial groups, increasing the aerobic/anaerobic bacterial ratio and, probably, impairing its balance. BT episodes in treated animals increased similarly to this ratio and were more frequent in animals with higher aerobic/anaerobic bacterial ratios. Of interest, total numbers of *Enterobacteriaceae* were increased in mice showing translocation of *E. coli* to MLNs, although differences did not reach statistical significance. Prevalence of each particular translocating species in MLNs could not be quantified in the corresponding gut sample, since number of gene copies was found below our detection limit (10^3^ copies) in all cases (data not shown).

Symbiotic mutualistic interactions between gut microbiota and the host are delineated at several physiological levels. Gut microbiota, for example, fulfils metabolic roles such as generation of short-chain fatty acids (SCFAs) that constitute an energy source, particularly for the host colonocytes [21]. Of the main SCFAs generated in the gut, butyrate also has the ability of suppressing inflammatory cytokine secretion by blocking NF-kB [22]. Although butyrate is mainly used at gut epithelium, polymorphnuclear (PMN) cells express a specific receptor for butyrate (GPR43) and other SCFAs like propionate and can be involved in butyrate signalling [23]. Although butyrate was not directly measured, the dysbiosis observed in our study was also associated with a decreased gene expression of its specific receptor GPR43 in neutrophils of treated mice. Since butyrate is mainly produced by species of the *Clostridium* clusters analysed such as the cluster XIV (*C. coccoides* group) and cluster IV (*C. leptum* group) that included *Faecalibacterium prausnitzii,* one of the main intestinal butyrate producer bacterium [24], and is implicated in the host's anti-inflammatory response, an enhanced pro-inflammatory response might be facilitated as a consequence of *Clostridium* groups decreased counts. In agreement with that, pro-inflammatory cytokines TNF-α and IL-6 were significantly increased in serum of treated mice and nicely correlated with the decrease of numbers of these *Clostridium* clusters and the GPR43 expression (Figures 3 and 4). GPR43 also participates in recruitment of other PMNs to prevent BT [23]. Accordingly, when *Clostridium* group numbers were reduced, GPR43 expression was down-regulated and pro-inflammatory cytokines were secreted, BT episodes were increased in treated animals, correlating with the aerobic/anaerobic bacterial ratio increase. In fact, BT has been associated with aerobic bacterial overgrowth [17]. All these findings suggest a role for microbiota changes in the development of BT episodes in treated mice.

**Figure 5:**
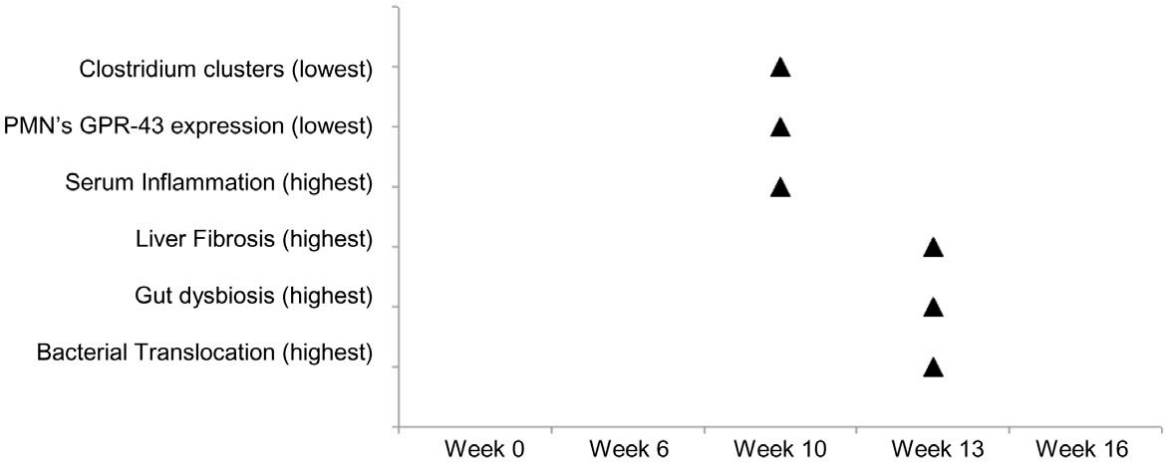
Temporal pattern of key events related with development of bacterial DNA translocation in CCl_4_-treated mice. The lowest levels of cytokine production, PMN butyrate specific-receptor GPR43 and *Clostridium* clusters counts from samples of intestinal cecum content along with the highest degrees of liver fibrosis, gut dysbiosis and bacterial translocation observed in treated animals are chronologically represented during the 16 weeks of study protocol.

It has been largely discussed whether BT induces inflammation or is facilitated by a pro-inflammatory environment [12]. While both probably feedback each other, in this model of liver damage, inflammation seems to be an early event in response to injure, likely as a consequence of a decrease in Gram-positive anaerobic *Clostridium* group numbers. This fact might help in providing the favourable conditions for fibrosis progression and BT. In fact, TNF-α, a key factor associated with fibrosis progression in many different types of chronic liver diseases [25], [26], is also associated with development of BT in rats subjected to induced liver damage with CCl_4_, hemorrhagic shock or with injection of endotoxin [12], [27]-[29]. Figure 5 resumes a list of events along the weeks of the study protocol in treated animals.

In summary, besides the important role that gut microbiota plays in the pathogenesis of complications in established cirrhosis such as bacterial infections, mainly caused by translocation of enteric Gram-negative microorganisms [19], we here suggest a role for alterations in intestinal microbiota composition in the development of an inflammatory outlook that may favour BT during early stages and progression of fibrosis. Although we cannot exclude the possibility that the altered gut flora composition was a result of CCl_4_ administration, these results enhance the relevance of promoting gut homeostasis to prevent adverse events such as BT in the setting of chronic liver damage.

## Methods

### Ethics statement

Animals received care according to the criteria outlined in the Guide for the Care and Use of Laboratory Animals. The study was approved by the Animal Research Committee of Universidad Miguel Hernandez (Alicante, Spain) with approval number HA-RFG-002-09.

### Animals and induction of liver damage

Female Balb/c mice (Harlan, Barcelona, Spain) were included in a 16-week study protocol. Mice were caged at a constant room temperature of 21°C and exposed to a 12∶12 light/dark cycle. Mice weighting 18–20 g were fed standard rodent chow and were treated with 0.25 mmol/L phenobarbital in tap water along the study protocol. After a 4-week housing in those conditions, animals received two weekly doses of CCl_4_ (Sigma Aldrich, Madrid, Spain) that were intragastrically administered using a sterile pyrogen free syringe with an attached stainless steel animal feeding tube without anaesthesia. The first dose of CCl_4_ was (100 uL/kg) in mineral oil, and subsequent doses were adjusted based on changes in weight 48 hours after the last dose. A second group of animals were used as controls. This group was fed standard rodent chow and treated with 0.25 mmol/L phenobarbital in tap water along the study protocol, and received two weekly doses of mineral oil (0.2 mL).

### Laparotomy and sample collection

Laparotomies were performed at 6, 10, 13 and 16 weeks in a subgroup of treated mice (n = 6/week) and at 0, 6, 10, 13 and 16 weeks in a subgroup of control animals (n = 4/week). Laparotomies were performed under anaesthesia with isofluorane. Abdominal fur was removed with a depilatory, and the skin was sterilized with iodine. A short incision in the abdominal wall was performed and all detectable mesenteric lymphatic nodes (MLNs), especially from the ileo-cecal area, were aseptically dissected and removed. Animals under anaesthesia were then euthanized by heart injection and total blood collection. Liver, spleen and intestinal content from ileum (10cm) and cecum were collected. Histopathological, microbiological and molecular studies were performed in all collected samples.

### Histological analysis

Forty-six liver biopsy specimens between 10 to 15 mm in size were fixed in buffered formalin and embedded in paraffin. Histologic changes were first evaluated by routine hematoxilin and eosin (H&E) in four-micrometer thick sections. We estimated the severity of hepatic fibrosis and architectural distortion with the connective tissue stain Masson trichrome. The amount of fibrosis was blindly assessed semiquantitatively based on the Ishak score [30] by a single senior pathologist (GP), with a conventional light microscope (Olympus BX50, Barcelona, Spain).

### Gene expression analysis

Total cellular RNA was isolated from 20–30 mg of liver disrupted by sonication (Hielscher UP100H Ultrasonic Processor, Teltow, Germany) to determine expression of profibrogenic tumour growth factor beta (TGF-β)-1, Procollagen α-1(1) (ProCol-1), tissue inhibitor of metalloproteinase (TIMP)-1 and matrix metalloproteinase (MMP)-2 genes and from 5×10^6^ blood polymorphonuclear (PMN) cells by handling QIAmp RNA Blood Minikit (QIAgen, Hilden, Germany) for quantitation of G Protein-coupled Receptor (GPR)-43, a specific butyrate-receptor gene. Quantitec SYBR Green (QIAgen) was used to perform gene expression in an IQ5 Real-Time PCR (BioRad, Hercules, CA). Table 4 resumes primer pair sequences used in the study. Primer-BLAST tool from NCBI (National Center for Biotechnology Information, U.S. National Library of Medicine) was used to design specific primers for studied profibrogenic genes.

**Table 4:** List of primer pairs used in the study.

Gene	Sequence (5′–3′)	References
β-2 microglobulin	GTGACCCTGGTCTTTCTGGT	
	ATCCCAGTAGACGGTCTTGG	
Procollagen α1 type I	TCCGGCTCCTGCTCCTCTTA	
	GTATGCAGCTGACTTCAGGGATGT	
Matrix metalloproteinase (MMP)-2	CCGAGGACTATGACCGGGATAA	
	CTTGTTGCCCAGGAAAGTGAAG	
Tumour growth factor (TGF)-β	CCTGAGTGGCTGTCTTTTGA	
	CAACCCAGGTCCTTCCTAAA	
Tissue inhibitor of metalloproteinase (TIMP)-1	TCCTCTTGTTGCTATCACTGATAGCTT	
	CGCTGGTATAAGGTGGTCTCGTT	
16S rRNA	AGAGTTTGATCATGGCTCAG	[13]
	ACCGCGACTGCTGCTGGCAC	
G protein-coupled receptor (GPR) 43	CCAAGGAGTTCTGGCAGGTGGCT	[32]
	AGCGCGCACACGATCTTTGGT	
***Target bacterial group/species***		
Total bacteria	TGGCTCAGGACGAACGCTGGCGGC	[33]
	CCTACTGCTGCCTCCCGTAGGAGT	
*Bacteroides fragilis group*	ATAGCCTTTCGAAAGRAAGAT	[33], [34]
	CCAGTATCAACTGCAATTTTA	
*Clostridium coccoides group*	AAATGACGGTACCTGACTAA	[33], [34]
	CTTTGAGTTTCATTCTTGCGAA	
*Clostridium leptum group*	GCACAAGCAGTGGAGT	[33], [34]
	CTTCCTCCGTTTTGTCAA	
*Enterobacteriaceae*	CATTGACGTTACCCGCAGAAGAAGC	[35]
	CTCTACGAGACTCAAGCTTGC	
*Lactobacillus group*	GGAAACAG(A/G)TGCTAATACCG	[36], [37]
	CACCGCTACACATGGAG	
*Bifidobacterium*	CTCCTGGAAACGGGTGG	[33], [34]
	GGTGTTCTTCCCGATATCTACA	
*E. coli*	GTTAATACCTTTGCTCATTGA	[35]
	ACCAGGGTATCTAATCCTGTT	
*K. pneumoniae*	TGAAATATGACTCCACTCACGG	
	CTTCAGAAGCGGCTTTGATGGCTT	
*Enterococcus spp.*	CCCTTATTGTTAGTTGCCATCATT	[35]
	ACTCGTTGTACTTCCCATTGT	
*S. pneumoniae*	AAGGAGGGGTGTGTAC	[38]
	GTACAACGAGTCGCAAGC	
*S. aureus*	GGCCGTGTTGAACGTGGTCAAATCA	[39]
	TACCATTTCAGTACCTTCTGGTAA	
*Shigella group*	GTTCCTTGACCGCCTTTCCGATAC	[40]
	CATTTCCTTCACGGCAGTGGA	
*C. jejuni*	GAAGAGGGTTTGGGTGGTG	[41]
	AGCTAGCTTCGCATAATAACTTG	

### Identification of bacterial DNA

Bacterial DNA presence was detected and identified in blood and MLNs. Extraction of total DNA was carried out by using the QIAamp DNA blood Mini kit (QIAgen), according to the manufacturer's instructions. A broad-range polymerase chain reaction (PCR) followed by nucleotide sequencing of 16SrRNA gene was performed according to the methodology described elsewhere [31]. Species-specific primers (Table 4) were also used to test simultaneous presence of several species by conventional PCR.

### Microbial composition analysis by quantitative PCR (qPCR)

Samples of intestinal content from cecum were frozen immediately after collection at -20°C and stored until analyzed. Samples were weighed and diluted 1∶5 (wt/vol) in phosphate-buffered saline (pH 7.2) homogenized and one aliquot of this dilution was used for DNA extraction. Extraction of total DNA from fecal samples was carried out by using the QIAamp DNA stool Mini kit (QIAgen) according to the manufacturer's instructions. The DNA obtained in the samples was measured with a ND-1000 spectrophotometer based absorbance at 260 nm (NanoDrop, Wilmington, DE).

Specific primers (Table 4) targeting different bacterial genera were used to characterize the main gut bacterial groups by quantitative real-time PCR. PCR amplification and detection were performed with LightCycler®480 Real-Time PCR System (Roche, Barcelona, Spain). Each reaction mixture (15 µL) consisted of LightCycler® 480 SYBR Green I Master (Roche), 0.75 µL of each of the specific primers at a concentration of 10 µM, and 60 ng of template DNA. The fluorescent products were detected at the last step of each cycle. A melting curve analysis was made after amplification to distinguish the targeted PCR product from the non-targeted PCR product. Bacterial concentration from each sample was calculated by comparing the Ct values obtained from the standard curves. Standard curves were created using serial 10-fold dilution of pure cultures of DNA, corresponding to 10^2^ to 10^9^ cells from the culture collection, as determined by microscopy counts using DAPI.

### Serum Cytokine Levels

Enzyme-linked immunosorbent assays (ELISAs) were carried out for IL-6 and TNF-α (Human Quantikine kits, R&D Systems, Minneapolis, MN) according to the manufacturers' instructions. All samples were tested in triplicate and read in a Sunrise Microplate Reader (Tecan, Männedorf, Switzerland). Lower limit of detection for each assay was 5 pg/mL. Standard curves were generated for every plate and the average zero standard optical densities were subtracted from the rest of the standards, controls, and samples to obtain a corrected concentration.

### Statistical analysis

Continuous variables are reported as mean ± standard deviation and categorical variables as frequency or percentages. Statistical differences of basal characteristics between groups were analyzed using the Chi test for categorical data and the Mann-Whitney U test for quantitative data. Bivariate correlations between continuous variables were calculated using the Spearman test. All reported *P* values are 2-sided, and *P* values lower than 0.05 were considered to indicate significance. All calculations were performed using the SPSS 16.0 software (SPSS, Inc, Chicago, IL).
